# Supplementary material for: Interface between Bats and Pigs in Heavy Pig Production
Source: Viruses. 2020 Dec 22;13(1):4. doi: 10.3390/v13010004 (PMC7822039; doi:10.3390/v13010004)

Supplemental material

Supplementary methods

Bioacoustic analyses

Bioacoustics techniques rely on the recording and analysis of the echolocation calls that all European species produce for orientation and prey detection, consisting in sonar signals generated in the larynx and emitted through either the mouth or the nostrils (Fenton et al., 2016). Bat acoustic signals are characteristic for each bat genus and, sometimes, for each bat species, allowing to monitor the biodiversity of chiropters based on bioacoustics only. However, some bats are able to modulate their emissions to match different habitat structure or allow specific activities, such as open flight or foraging (Schnitzler and Kalko, 2001). This plasticity of bat calls leads to a certain overlapping between sonograms of different species and strongly challenges the use of algorithms for automatic identification in favor of the intervention of a chiropterologist with expertise on bioacoustics (Russo and Voigt, 2016).

For the purpose of this study we used bioacoustics to identify the circulating bat species and to quantify and characterize the activity of bats in each farm. In order to do this, we determined the host species of each bat call recorded with the bat detector and we calculated the following parameters:

- *Bat activity:* number of bat passes per night, determined over an average of 12 recording days;
- *Species richness*: total number of bat species recorded on each farm;
- *Species activity*: number of bat passes per hour, referred to each species and reported as the mean value calculated over an average of 12 recording days;
- *Species occurrence*: percentage of sounds to be ascribed to each single species over the total passes;
- *Feeding activity*: also called buzz-ratio, defined as the percentage of feeding buzzes over the total passes occurring in each farm per recording night. This parameter was determined both for bats in general and for each bat species found within the farm.
- *Social activity:* percentage of social calls over the total passes occurring in each farm per recording night. This parameter was determined both for bats in general and for each bat species found within the farm.

According to these parameters, the activity of bats within each farm was described using the following categories:

- Passage: Sole recording of echolocation calls typical of open flight, with neither social calls nor feeding buzzes. The passage was further divided in rare (*activity* < 1.5p/h e *occurrence* < 5), occasional (*activity* >1.6% or *occurrence* >6%) or habitual (*activity* >10% or *occurrence* >25%)
- Foraging: recording of feeding buzzes with or without social calls. The foraging activity was further divided into occasional feeding (*feeding activity* < 10%), feeding zone (*feeding activity* > 10%) and habitual feeding zone (*feeding activity* > 10% associated with the recording of social calls)
- Roosting: recording of echolocation calls, including social calls, soon after sunset. The category showing roosting activity for pig farms is likely to include several cases in which the actual roost is located in the proximity of the farm rather than within its perimeter.

Visualization of tanglegrams for the analysis of phylogenetic incongruence

We used an in-house pipeline developed at our institute for creating the tanglegram showed as Figure 5. The pipeline is composed of a C++ code that implements some heuristics processes to minimize the number of intersections between trees, followed by an R script that generates the figure. Briefly, each input tree in newick format is converted into a graph composed of nodes and branches. Nodes are sorted in alphabetical order using their names, which are assigned by default if not present in the input data. Following such order, for each node all its possible rotations are computed. Then all rotations of all nodes belonging to a tree are combined together, up to a maximum amount which can be adjusted with parameter "-rotate_trees". Combinations that minimize the number of intersections with respect the previous tree are stored, up to a maximum amount which can be adjusted with parameter "-max_trees". This operation is performed for each tree starting from the second one given in input; generating a list of which a single element is a particular combination of exactly one rotation of all input trees. Such list is stored up to a maximum amount which can be adjusted with parameter "-save_trees". To sum up, the implemented heuristic model searches a local minimum value among the landscape of all the possible number of intersections; the search can be wider using a larger value for the 3 previous parameters mentioned, at the cost of more computation time a RAM memory. Currently the pipeline is available only on request and a manuscript is in preparation.

Supplementary Tables

Table S1. Primers used in the study to elongate BtCoV/19RS-495-11/P.kuhlii/ITA/2018.

Name	Sequence 3’...5’	Tm °C	Use
RdRp_BCoV_13183F	TGCATTGTGCCAATTTCAACAC	56.5	Amplification and sequencing
RdRp_BCoV_14292-R	GAATTAGCATATGCAGTAGTAGC	57.1	Amplification and sequencing
RdRp_BCoV_13620F	GATTTTTACCGGTACAATAGGC	56.5	Sequencing
RdRp_BCoV_13872R	TCCTTACCACTAATGGCATACTT	57.1	Sequencing

Primers were designed based on the alignment of two Italian sequences of CoVs described from *Pipistrellus kuhlii* (BtCoV/KF500949/P.kuhlii/Italy/2010 and BtCoV/MH938450/P.kuhlii/Italy/2010), showing 98.2% ID at the nucleotidic level.

Table S2. Nucleotidic distance between MRV from bats and pigs found in Farm 1 between 2016 and 2018

	L1	L2	L3	M2	M3	S1	S2	S3	S4
Nucleotidic p-distance (SE)									
Myotis vs swine 2018	13.1 (0.007)	24 (0.012)	15.7 (0.006)	23.2 (0.013)	19 (0.008)	38.5 (0.014)	15.6 (0.01)	13.3 (0.01)	9.5 (0.009)
Myotis vs swine 2016	13 (0.007)	24.2 (0.012)	15.6 (0.006)	23.3 (0.012)	18.3 (0.008)	60.6 (0.014)	15.7 (0.011)	13.3 (0.01)	11.8 (0.01)
Amminoacidic p-distance (SE)									
Myotis vs swine 2018	1.9 (0.004)	6.7 (0.008)	0.008 (0.003)	6.6 (0.013)	8.3 (0.01)	40.5 (0.021)	2 (0.007)	3.6 (0.009)	5.1 (0.012)
Myotis vs swine 2016	1.8 (0.004)	7 (0.008)	0.008 (0.003)	4.4 (0.011)	8.2 (0.011)	74.7 (0.032)	2 (0.007)	3.9 (0.01)	9.3 (0.015)

Swine 2016: MRV3/16DIA52154-4/swine/Italy/2016; Swine 2018: 18DIA90178-3/swine/Italy/2018; *Myotis*: 18RS2900-2/*M.nattereri*/Italy/2018

Supplementary figures

Figure S1. Map showing the location of study farms across north eastern Italy.

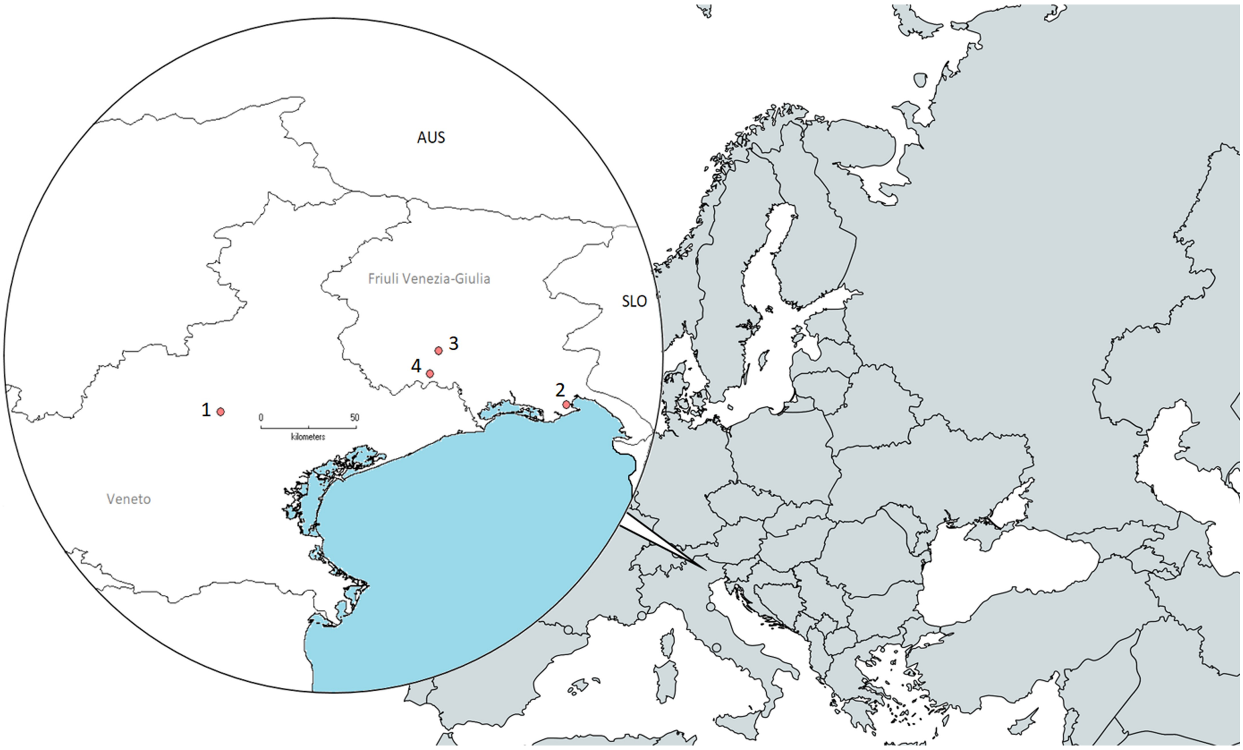

Supplement: Supplementary file 1 [file viruses-13-00004-s001.pdf]
